# The different risk factors for isolated diastolic hypertension and isolated systolic hypertension: a national survey

**DOI:** 10.1186/s12889-021-11686-9

**Published:** 2021-09-14

**Authors:** Kun Xie, Xiufang Gao, Liwen Bao, Ying Shan, Haiming Shi, Yong Li

**Affiliations:** Cardiology Department, Huashan Hospital, Fudan University, No. 12 Mid Wulumuqi Road, Shanghai, 200040 China

**Keywords:** Isolated diastolic hypertension, Aging, Body mass index, Smoking, Isolated systolic hypertension

## Abstract

**Background:**

Hypertension is highly prevalent and is one of the modifiable risk factors for cardiovascular outcomes. Isolated diastolic hypertension (IDH), however, tends to be ignored due to insufficient recognition. We sought to depict the clinical manifestation of IDH and isolated systolic hypertension (ISH) to find a more efficient way to improve the management.

**Methods:**

Patients with primary hypertension aged over 18 years were investigated from all over the country using convenience sampling during 2017–2019. IDH was defined as systolic blood pressure (SBP) < 140 mmHg and diastolic blood pressure (DBP) ≥90 mmHg. ISH was defined as SBP ≥ 140 mmHg and DBP < 90 mmHg.

**Results:**

A total of 8548 patients were screened, and 8475 participants were included. The average age was 63.67 ± 12.78 years, and males accounted for 54.4%. Among them, 361 (4.3%) had IDH, and 2096 had ISH (24.7%). Patients with IDH (54.84 ± 13.21 years) were much younger. Aging turned out to be negatively associated with IDH but positively associated with ISH. Multivariate logistic regression analysis showed BMI was a significant risk factor for IDH (OR 1.30, 95%CI 1.05–1.61, *p* = 0.018), but not for ISH (OR 1.05, 95%CI 0.95–1.16, *p* = 0.358). Moreover, smoking was significantly associated with IDH (OR 1.36, 95%CI 1.04–1.78, *p* = 0.026) but not with ISH (OR 1.04, 95%CI 0.90–1.21, *p* = 0.653).

**Conclusions:**

Patients with IDH were much younger, and the prevalence decreased with aging. BMI and smoking were remarkably associated with IDH rather than ISH. Keeping fit and giving up smoking might be particularly efficient in the management of young patients with IDH.

**Trial registration:**

NCT03862183, retrospectively registered on March 5, 2019.

## Background

Hypertension is highly prevalent and is one of the most modifiable risk factors for cardiovascular mortality and morbidity. Isolated diastolic hypertension (IDH) and isolated systolic hypertension (ISH) are two particular types of hypertension. However, compared to ISH, IDH tend to be ignored either by patients or by physicians. As reported in the PEACE Study, 86.1% of those IDH were untreated [1].

Recently, the significance of IDH has been challenged. McEvoy et al. reported that IDH, by 2017 ACC/AHA definitions [2], was not associated with increased cardiovascular outcomes [3]. That study might be limited by the population’s age, and the results should be cautiously generalized to young patients. Yue et al. found that in 21,441 participants, patients aged 35–59 years with stage 1 hypertension defined by the 2017 ACC/AHA guideline had a significantly increased risk of CVD risks over a 15-year period. However, in patients aged ≥60 years, stage 1 hypertension was not associated with increased CVD risks [4].

Actually, in a recent network meta-analysis, each 10 mmHg reduction in systolic BP and each 5 mmHg reduction in diastolic BP have been reported to be associated with a lower risk of cardiovascular mortality, cardiovascular events, and stroke [5]. Lee et al. reported that among 6 million participants aged 20–39 years, stage 1 IDH turned out to be associated with higher CVD risks after 13.2 years follow up [6]. Thus, the management of IDH in young patients should be highlighted rather than ignored.

However, adherence to antihypertensive medication is far from optimal, especially in younger patients. Tiffany et al. reported that among 23.8 million hypertensive adults, the nonadherence rate was around 31%, and the highest nonadherence rate of 58.1% was seen in the youngest population aged 18–34 years in the U.S. in 2015 [7].

Besides antihypertensive drugs, lifestyle management also plays an essential role in managing hypertension concerning the high nonadherence rate in young patients [8–10]. Nevertheless, lifestyle management may affect different types of hypertension like IDH and ISH, which are considered to have different pathophysiological mechanisms and will result in various features [11]. However, our knowledge of IDH is still insufficient, as is stated in the guidelines [2, 12]. We sought to depict the prevalence and clinical manifestation of IDH, ISH, systolic and diastolic hypertension (SDH), and normotension in a multicenter observational study (UPPDATE Study) to find a more efficient way to improve the management of IDH.

## Methods

We carried out a nationwide cross-sectional study (UPPDATE study, Survey on BP, Lipids and cardiovascular risks in the outpatient hypertensives and Continuing Medical Education Program) in hospitals all over the country from 2017 to 2019 using convenience sampling. These hospitals were located in the three municipalities directly under the Central Government (Beijing, Shanghai, and Chongqing) and 14 provinces all over the country (Table 1). Patients with hypertension, either under lifestyle management or antihypertensive medication, were included. The inclusion criteria were 1) ≥ 18 years old; 2) primary hypertension; 3) providing the written informed consent. The exclusion criteria were 1) secondary hypertension, 2) severe liver or renal disease, 3) mental illness, or active cancer. The research protocol was approved by the Ethics Committee of Huashan Hospital, Fudan University (2017–282-1). Age categories were defined as < 25 years, > 85 years, and every 10-year apart from 25 to 85 years. Hypertension was defined according to the 2018 ESC/ESH guideline for the management of hypertension using office BP [12]. Therefore, IDH was defined as SBP < 140 mmHg and DBP ≥ 90 mmHg; ISH was defined as SBP ≥ 140 mmHg and DBP < 90 mmHg; Systolic and diastolic hypertension (SDH) was defined as SBP ≥ 140 mmHg and DBP ≥ 90 mmHg; Normotension was defined as SBP < 140 mmHg and DBP < 90 mmHg. Elevated BMI was defined as overweight and obesity (BMI ≥ 25 kg/m^2^) according to the 2000 WHO standard [13]. The smoking history was defined as those who had a history of smoking, whether stopped or not. Patients with antihypertensive agents were defined as those under the treatment of any antihypertensive drugs recommended by the guidelines [12]. Predictor variables were selected based on the guidelines of hypertension and previous literature [12, 14, 15].

**Table 1:** Geological distribution of hospitals in UPPDATE Study (ranked by the number of hospitals in each municipal city or province)

Municipal city/Province	Number of hospitals (%)
**Shandong Province**	**21 (12.73%)**
**Zhejiang Province**	**17 (10.30%)**
**Jiangsu Province**	**15 (9.09%)**
**Shanghai**	**14 (8.48%)**
**Guangdong Province**	**11 (6.67%)**
**Hunan Province**	**11 (6.67%)**
**Liaoning Province**	**11 (6.67%)**
**Anhui Province**	**10 (6.06%)**
**Heilongjiang Province**	**9 (5.45%)**
**Yunnan Province**	**9 (5.45%)**
**Hubei Province**	**8 (4.85%)**
**Chongqing**	**8 (4.85%)**
**Shanxi Province**	**5 (3.03%)**
**Beijing**	**4 (2.42%)**
**Guizhou Province**	**4 (2.42%)**
**Hebei Province**	**4 (2.42%)**
**Sichuan Province**	**4 (2.42%)**

Statistical analysis was conducted using STATA 13.1. Two-sides Student’s t-test or ANOVA were used for continuous variables, and Chi-square test was used for categorical variables. Both the forward stepwise method and the backward stepwise method were used to reach the multivariate logistic regression model. Variables with *p* < 0.25 in univariable logistic regression analysis would be included to build the model. *P* < 0.05 was considered statistically significant.

## Results

A total of 8548 participants were screened, and 73 of them were excluded due to missing covariates. Finally, 8475 participants were included. The average age was 63.67 ± 12.78 years. Males accounted for 54.4%. 18.5% of them had a habit of smoking, and 89.4% were under antihypertensive agents. The average BMI was 24.83 ± 3.85 kg/m^2^ (Table 2).

**Table 2:** Demographic characters of the total patients and patients in different BP categories

	Total (*n* = 8475)	Normotension (*n* = 3434)	IDH (*n* = 361)	ISH (*n* = 2096)	SDH (*n* = 2584)	p
Age (yrs)	63.67 ± 12.78	65.11 ± 11.90	54.84 ± 13.21	66.87 ± 12.09	60.39 ± 13.12	< 0.001
Male (%)	4610 (54.4%)	1820 (53.0%)	233 (64.5%)	1062 (50.7%)	1495 (57.9%)	< 0.001
BMI (kg/m^2^)	24.83 ± 3.85	24.50 ± 3.38	26.47 ± 6.53	24.70 ± 3.77	25.15 ± 3.92	< 0.001
Smoking (%)	1560 (18.5%)	538 (15.8%)	98 (27.4%)	354 (17.1%)	570 (22.2%)	< 0.001
Antihypertensive agents (%)	7567 (89.4%)	3264 (95.1%)	296 (82.0%)	1852 (88.4%)	2155 (83.5%)	< 0.001
SBP (mmHg)	142.50 ± 18.66	127.78 ± 8.06	126.72 ± 14.43	150.01 ± 11.52	158.17 ± 17.47	< 0.001
DBP (mmHg)	84.54 ± 12.73	76.43 ± 6.82	97.89 ± 14.40	79.17 ± 6.52	97.79 ± 9.76	< 0.001
LDL (mmol/L)	2.86 ± 1.09	2.74 ± 1.03	3.07 ± 1.45	2.87 ± 1.06	3.03 ± 1.13	< 0.001
TC (mmol/L)	4.86 ± 1.34	4.73 ± 1.26	4.94 ± 1.19	4.78 ± 1.26	5.14 ± 1.51	< 0.001
HDL (mmol/L)	1.28 ± 0.34	1.29 ± 0.35	1.23 ± 0.33	1.29 ± 0.33	1.27 ± 0.32	0.06
TG (mmol/L)	2.06 ± 1.67	1.94 ± 1.54	2.35 ± 1.74	1.91 ± 1.41	2.36 ± 2.01	< 0.001
DM (%)	1634 (20.2%)	709 (21.6%)	56 (15.9%)	473 (23.7%)	396 (16.2%)	< 0.001
CAD (%)	1435 (17.8%)	541 (16.5%)	42 (11.9%)	417 (21.0%)	435 (17.8%)	< 0.001
HF (%)	179 (2.2%)	64 (2.0%)	4 (1.1%)	57 (2.9%)	54 (2.2%)	0.07
Statin (%)	4461 (57.6%)	1834 (58.2%)	158 (46.1%)	1210 (64.3%)	1259 (53.2%)	< 0.001

BP: blood pressure. IDH: isolated diastolic hypertension. ISH: isolated systolic hypertension

*P*-values refer for ANOVA test for continuous variables and Chi-square test for dichotomous variables

Among them, 361(4.3%) patients had IDH, 2096 (24.7%) patients had ISH, 2584 (30.5%) patients had SDH, and 3434 (40.5%) patients had normotension. Patients with IDH were the youngest (54.84 ± 13.21 years) among those with ISH (66.87 ± 12.09 years), SDH (60.39 ± 13.12 years), and Normotension (65.11 ± 11.90 years). However, patients with IDH had the highest BMI (26.47 ± 6.53 kg/m^2^) among those with ISH (24.70 ± 3.77 kg/m^2^), SDH (25.15 ± 3.92 kg/m^2^), and normotension (24.50 ± 3.38 kg/m^2^). The smoking rate was also the highest in patients with IDH (27.4%) among those with ISH (17.1%), SDH (22.2%), and normotension (15.8%). While antihypertensive treatment rate was the lowest in patients with IDH (82.0%) among those with ISH (88.4%), SDH (83.5%), and normotension (95.1%). Statin treatment rate was also the lowest in patients with IDH (46.1%) among those with ISH (64.3%), SDH (53.2%), and normotension (58.2%) (Table 2).

**Fig. 1:**
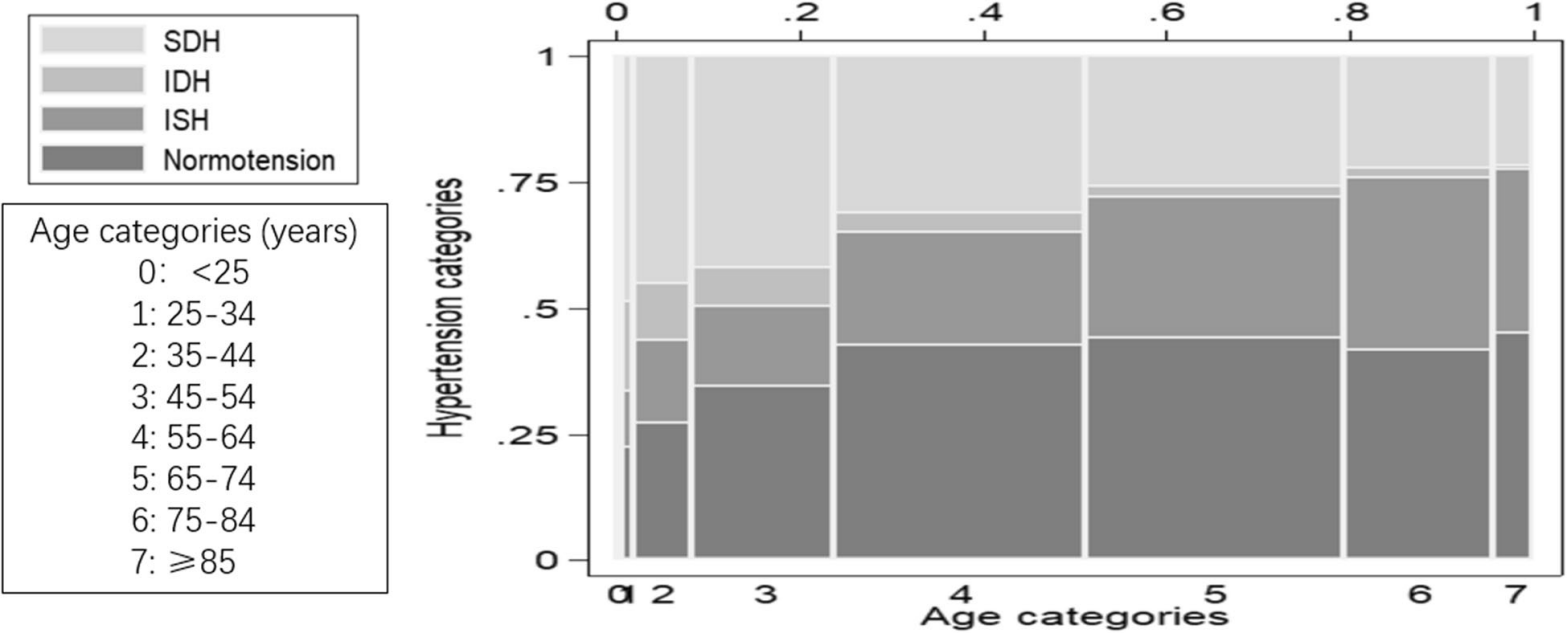
The proportion of IDH and ISH in patients with hypertension according to age categories/ IDH: isolated diastolic hypertension. ISH: isolated systolic hypertension. SDH: systolic and diastolic hypertension. Different dimensions along with the horizontal axis are related to sample proportions

**Fig. 2:**
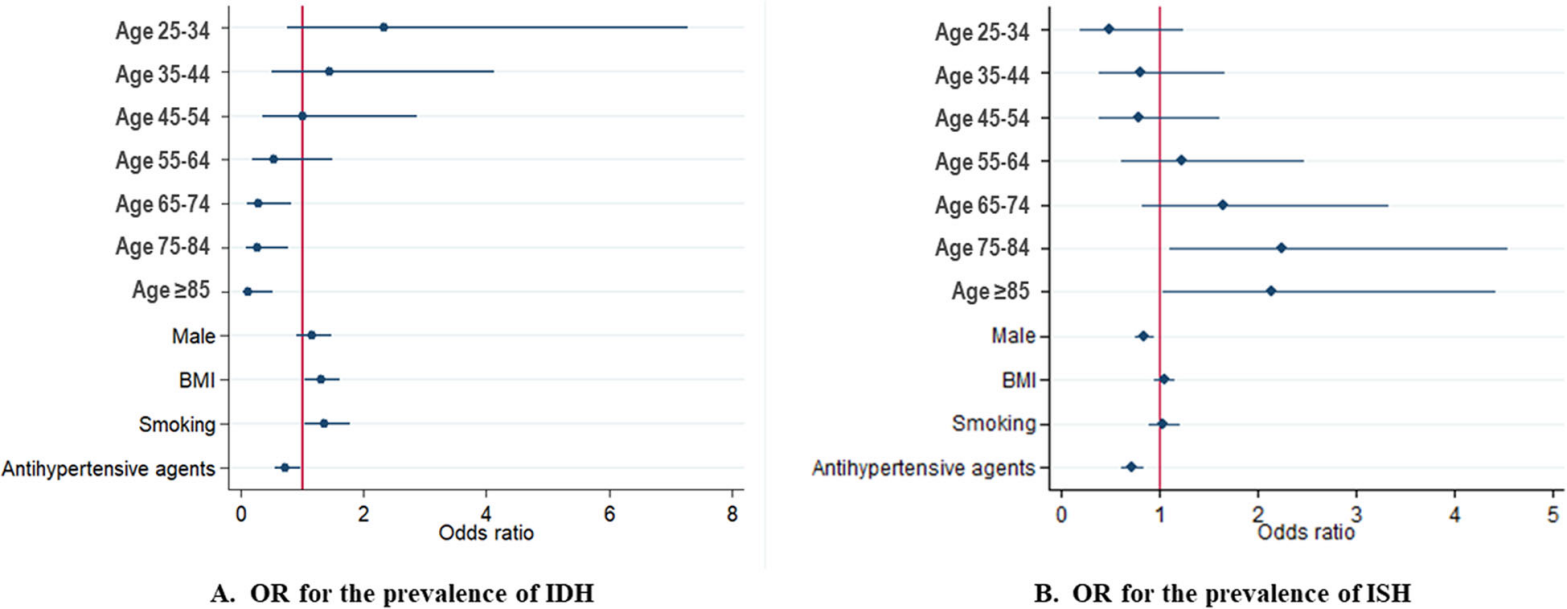
OR for the prevalence of IDH and ISH. A. OR for the prevalence of IDH; B. OR for the prevalence of ISH. IDH: isolated diastolic hypertension. ISH: isolated systolic hypertension

The proportion of IDH of the total population decreased with aging, while the proportion of ISH increased with aging (Table 3, Fig. 1). Logistic regression analysis showed the gradually decreasing risk of IDH and the continuously increasing risk of ISH for every 10-year aging, which means aging was positively associated with ISH but negatively associated with IDH (Table 4, Fig. 2). Younger patients were more likely to have IDH, while older patients were more likely to have ISH.

**Table 3:** The distribution of different types of hypertension according to age

Age categories (Years)	Normotension	IDH N (%)	ISH N (%)	SDH
< 25	23 (0.7)	4 (1.1)	10 (0.5)	14 (0.5)
25–34	24 (0.7)	19 (5.3)	12 (0.6)	52 (2.0)
35–44	151 (4.4)	63 (17.5)	90 (4.3)	249 (9.6)
45–54	452 (13.2)	101 (28.0)	210 (10.0)	550 (21.3)
55–64	986 (28.7)	93 (25.8)	516 (24.6)	716 (27.7)
65–74	1059 (30.8)	52 (14.4)	670 (32.0)	618 (23.9)
75–84	575 (16.7)	26 (7.2)	470 (22.4)	306 (11.8)
≥85	164 (4.8)	3 (0.8)	118 (5.6)	79 (3.1)
Total	3434 (100)	361 (100)	2096 (100)	2584 (100)

IDH: isolated diastolic hypertension. ISH: isolated systolic hypertension

**Table 4:** Trends in the OR for the prevalence of IDH or ISH

	Model 1		Model 2		Model 3	
	OR (95%CI)	p	OR (95%CI)	p	OR (95%CI)	p
**Prevalence of IDH**						
Age						
< 25	Reference	–	Reference	–	Reference	–
25–34	2.53 (0.82–7.89)	0.108	2.40 (0.77–7.49)	0.130	2.33 (0.75–7.29)	0.145
35–44	1.51 (0.53–4.33)	0.443	1.47 (0.51–4.22)	0.474	1.43 (0.50–4.12)	0.505
45–54	1.00 (0.35–2.77)	0.968	0.98 (0.34–2.77)	0.965	1.01 (0.36–2.87)	0.982
55–64	0.49 (0.17–1.40)	0.183	0.50 (0.18–1.42)	0.193	0.53 (0.18–1.50)	0.228
65–74	0.26 (0.09–0.75)	0.013	0.27 (0.09–0.77)	0.014	0.28 (0.10–0.82)	0.020
75–84	0.22 (0.08–0.67)	0.008	0.23 (0.08–0.69)	0.009	0.26 (0.09–0.77)	0.015
≥ 85	0.10 (0.02–0.45)	0.003	0.10 (0.02–0.45)	0.003	0.11 (0.02–0.52)	0.005
Male	1.55 (1.25–1.94)	< 0.001	1.32 (1.05–1.65)	0.015	1.16 (0.90–1.48)	0.249
Elevated BMI	1.55 (1.25–1.91)	< 0.001	1.34 (1.08–1.66)	0.008	1.30 (1.05–1.61)	0.018
Smoking	1.70 (1.34–2.16)	< 0.001	1.35 (1.03–1.76)	0.029	1.36 (1.04–1.78)	0.026
Antihypertensive Agents	0.52 (0.40–0.69)	< 0.001	0.76 (0.57–1.02)	0.063	0.73 (0.54–0.97)	0.031
**Prevalence of ISH**						
Age						
< 25	Reference	–	Reference	–	Reference	–
25–34	0.52 (0.21–1.29)	0.159	0.53 (0.21–1.33)	0.179	0.49 (0.19–1.24)	0.131
35–44	0.80 (0.39–1.65)	0.541	0.81 (0.39–1.67)	0.568	0.81 (0.39–1.67)	0.566
45–54	0.78 (0.38–1.58)	0.492	0.78 (0.39–1.58)	0.494	0.79 (0.39–1.61)	0.517
55–64	1.18 (0.59–2.37)	0.645	1.17 (0.58–2.35)	0.662	1.23 (0.61–2.48)	0.556
65–74	1.59 (0.79–3.19)	0.193	1.57 (0.78–3.15)	0.205	1.66 (0.82–3.34)	0.157
75–84	2.12 (1.05–4.28)	0.035	2.10 (1.04–4.23)	0.038	2.25 (1.11–4.54)	0.024
≥ 85	1.97 (0.95–4.06)	0.068	1.96 (0.95–4.04)	0.070	2.13 (1.03–4.42)	0.041
Male	0.82 (0.74–0.90)	< 0.001	0.87 (0.78–0.96)	0.005	0.85 (0.76–0.95)	0.003
Elevated BMI	0.98 (0.89–1.08)	0.693	1.05 (0.95–1.16)	0.330	1.05 (0.95–1.16)	0.358
Smoking	0.88 (0.77–1.00)	0.047	1.03 (0.89–1.19)	0.723	1.04 (0.90–1.21)	0.563
Antihypertensive Agents	0.88 (0.75–1.03)	0.113	0.73 (0.62–0.86)	< 0.001	0.72 (0.61–0.85)	< 0.001

Model 1 was unadjusted model. Model 2 was adjusted for age and gender. Model 3 was adjusted for elevated BMI, age categories, gender, smoking and antihypertensive agents

IDH: isolated diastolic hypertension. ISH: isolated systolic hypertension

Additionally, patients with IDH had a higher BMI (26.47 ± 6.53 kg/m^2^) than those with ISH (24.70 ± 3.77 kg/m^2^). In multivariate logistic regression analysis, after adjusting for age, gender, smoking history, and antihypertensive agents, BMI remained as a significant risk factor for IDH (OR 1.30, 95%CI 1.05–1.61, *p* = 0.018), but not for ISH (OR 1.05, 95%CI 0.95–1.16, *p* = 0.358), which means the BMI target has not been achieved well and remained to be taken seriously, particularly in patients with IDH (Table 4, Fig. 2).

Moreover, smoking was more prevalent in IDH (27.4%) than in ISH (17.1%). Multivariate logistic regression analysis showed that, after adjusting for age, gender, BMI, and antihypertensive agents, smoking remained significantly associated with IDH (OR 1.36, 95%CI 1.04–1.78, *p* = 0.026) but not with ISH (OR 1.04, 95%CI 0.90–1.21, *p* = 0.653). Therefore, besides BMI, stop smoking is another recommended lifestyle change that should be seriously concerned (Table 4, Fig. 2).

## Discussion

Our study found that IDH and ISH were two distinctive types of hypertension. Both of them were age-dependent. However, IDH was more prevalent in young and middle-aged patients, while ISH was more prevalent in middle-aged and old patients. Obesity and smoking, the risk factors for hypertension and lifestyle-changing targets, were significantly associated with IDH but not ISH. Therefore, BMI and smoking habits should be concerned more seriously and might be particularly efficient in young patients with IDH.

Recently, diastolic BP was not considered as important as systolic BP, and IDH was challenged to be regarded as the risk factor for incident cardiovascular outcomes by some studies [3, 16, 17]. Moreover, Mahajan et al. reported that few patients with IDH were aware of having hypertension and were poorly managed in the China PEACE Million Persons Project [1].

Nevertheless, in a worldwide study, Yan Li et al. reported that IDH was remarkably associated with cardiovascular events, particularly in those below 50 years [18]. Additionally, IDH was associated with urinary albumin/creatinine ratio, particularly in patients below 55 years [19]. The inconsistent results may mainly lie in the age of the study population. It has already been recognized that after 50 years old, the systolic BP continuously increases with age, while on the other hand, the diastolic BP starts to decrease with age. Therefore, IDH is more prevalent in young and middle-aged patients, but ISH is more prevalent in middle-aged and old patients [8, 20]. Thus, the management of IDH should be highlighted rather than ignored in younger patients [21–23].

Obesity and smoking are the two major risk factors for the development of hypertension, which can be modified by improving lifestyle management [2, 24]. As obesity and smoking are highly prevalent in young adults, it would be particularly essential to prevent cardiovascular disease by early lifestyle management. BMI trajectories are significantly associated with the incidence of hypertension in young adults, which suggested the importance of early prevention [25]. Recently, smoking has been confirmed to be associated with an increased risk of masked hypertension, especially in heavy smokers [26]. In our study, BMI was remarkably associated with the prevalence of IDH but was not associated with ISH, which might suggest that lowering BMI might be an effective way to lower diastolic BP and improve the management of IDH. Smoking was significantly associated with the prevalence of IDH as well. It was not associated with ISH, either. Thus, giving up smoking might also be exceptionally efficient in the management of IDH. More longitudinal studies are needed in the early IDH interference and management.

The weakness of our study was that it was a cross-sectional study with convenience sampling, which would cause selection bias. The results could not be generalized to those who never came to hospitals and we could not tell the causal relationship between BMI, smoking, and IDH. Patients with ISH were older and might have better lifestyle management after years of medical contact to have lower BMI and smoking rates. Moreover, as we know, patients with hypertension have different weight on risk factors. Other risk factors might affect more in patients with ISH. In addition, unmeasured factors like diet, physical activity levels, socio-economic status, educational levels, and the presence of HMOD were not included in the analysis, which would prevent the study from providing a whole picture of the characteristics of hypertensive patients and would also cause bias. However, we could find that more efforts were needed in lifestyle management concerning BMI and smoking in patients with IDH than those with ISH. Keeping fit and giving up smoking might be critical to lower the diastolic BP and to manage IDH.

The strength of our study was that it was a multi-center study recruiting patients from all over the country with a relatively large sample. Thus, it would be appropriate for the results to be generalized to the hypertensive patients who attend to hospitals in China. Additionally, our study population had a wide span of ages, which facilitated us to depict the features of IDH in relatively young patients and helped fill the insufficiency of data in patients with IDH. The result suggested the importance of lifestyle management in the early-onset patients with IDH, besides antihypertensive agents.

In conclusion, IDH and ISH had different features. Patients with IDH were much younger, and the prevalence decreased with aging. On the contrary, patients with ISH were much older, and the prevalence increased with aging. As IDH was a disease of young and middle-aged patients, such subtype management should be highlighted rather than ignored. BMI and smoking status were the two factors mainly associated with IDH rather than ISH. Besides antihypertensive agents, keeping fit and giving up smoking might contribute a lot to managing young patients with IDH.

## Data Availability

The datasets used and analyzed during the current study are available from the corresponding author on reasonable request.
